# Where does it come from, where does it go? The role of the xylem for plant CO_2_ efflux

**DOI:** 10.1093/jxb/erx161

**Published:** 2017-07-17

**Authors:** Arthur Gessler

**Affiliations:** Swiss Federal Research Institute WSL, Research Unit Forest Dynamics, Zuercherstr., Birmensdorf, Switzerland

**Keywords:** Assimilation, carbon isotope, environmental drivers, respiration, transpiration

## Abstract

This article comments on:

Stutz SS, Anderson J, Zulick R, Hanson DT. 2017. Inside out: efflux of carbon dioxide from leaves represents more than leaf metabolism. Journal of Experimental Botany 68, 2849–2857.

**Not that long ago, the CO_2_ efflux from a plant organ was attributed to metabolic reactions and activity only in that same organ. For tree stems, however, we know that CO_2_ transported in the xylem and originating from other plant compartments compromises stem respiration measurements. Now, Stutz *et al.* (2017) have shown that leaf CO_2_ efflux, in addition, does not solely represent leaf metabolic processes but is affected by xylem-borne CO_2_.**

Knowledge of the processes of carbon (C) allocation and cycling in plants and ecosystems is important for understanding the terrestrial C cycle as a whole and changes as a result of anthropogenic impacts. The potential of ecosystems to sequester C from the atmosphere reflects the balance between C assimilation and the complex set of respiration processes (Trumbore *et al.*, 2013). Even though respiration is likely to be the most important determinant of the C balance in terrestrial ecosystems (Valentini *et al.*, 2000), understanding of the mechanistic background is far from complete. On the one hand, respiration is affected by direct environmental cues that regulate respiratory metabolism (Atkin *et al.*, 2005). However, on the other hand, substrate supply for respiration, which is indirectly coupled to environmental drivers (e.g. via their effects on photosynthesis), controls respiratory CO_2_ fluxes (Högberg and Read, 2006). Besides photosynthesis, phloem transport and the partitioning of assimilates among different C pools affect substrate availability for respiration. We need greater acknowledgement that our view of the controls of respiration fluxes is incomplete (e.g. Hagedorn *et al.*, 2016) and that the ‘system plant’ might be more complex than previously thought.

## CO_2_ efflux from a given plant organ is not necessarily an indicator of its respiratory activity

Only recently, a major new challenge became obvious: CO_2_ efflux is not only affected by the catabolic metabolism of a given organ, but also by the respiration rate of plant compartments coupled via the transpiration stream to that organ. Thus, the CO_2_ exchange of stems, for example, is influenced by downstream CO_2_ production in the roots and the transpiration stream, adding within-plant spatial interconnectivities (Teskey *et al.*, 2008).

While it was previously usually assumed that CO_2_ produced in roots and stems escaped these organs more or less directly into the surrounding atmosphere, there is now strong evidence for trees that at least part of this CO_2_ remains inside the plant, gets dissolved in the xylem sap, and is then transported acropetally (Teskey and McGuire, 2002; McGuire and Teskey, 2004; Teskey *et al.*, 2008). Whilst parts of this xylem-transported CO_2_ escape from stems and branches into the atmosphere, up to almost 20% can be re-assimilated, with the highest contribution made by woody branches (Bloemen *et al.*, 2013).

Using a ^13^C-labelling approach, Stutz *et al.* (2017) have now shown that CO_2_ originating from the xylem also contributes to leaf CO_2_ efflux, and they were also able to quantify the contribution of xylem-originating CO_2_ to this flux (Box 1). Moreover, they determined the retention of CO_2_ in the leaf as a result of anaplerotic processes in the dark. While there are now a number of papers reporting analyses of the effect of xylem transport of CO_2_ on stem or branch efflux (e.g. Bowman *et al.*, 2005; Gansert and Burgdorf, 2005; Teskey and McGuire, 2005; Maier and Clinton, 2006), there is a lack of information on its contribution to ‘leaf respiration’. There is one observation by Stringer and Kimmerer (1993) showing that over 99% of xylem-transported CO_2_ is fixed in the light, whereas 80% of the transpired label escaped the leaves in the dark. For a whole tree, based on a mass balance approach, Bloemen *et al.* (2013) calculated that about 90% of the xylem CO_2_ left the tree via the stem and branches before reaching the leaves.

Stutz *et al.* (2017) related leaf efflux of xylem CO_2_ (^13^C-labelled) to ‘real’ leaf respiration (non-labelled) and showed that flux can approach 50% of the latter depending on transpiration rate and xylem CO_2_ concentrations. By taking the effects of light-enhanced dark respiration into account – an experimental artefact occurring when light-acclimated leaves are transferred into darkness and characterized by a burst of respiration exceeding ordinary dark respiration (Azcon-Bieto and Osmond, 1983) – the authors determined a realistic relationship between leaf respiratory CO_2_ flux and the CO_2_ efflux related to CO_2_ transported in the xylem.

**Box 1. CO_2_ fluxes in plant stems and leaves**

Up to 50% of root-respired CO_2_ is transferred via the xylem stream aboveground (Bloemen *et al.*, 2013) and reaches the stem sapwood (in woody plants). Part of this CO_2_ escapes from the stem to the atmosphere, whilst part is assimilated via Rubisco in the photosynthetically active bark and via phosphoenolpyruvate carboxylase. Moreover, (net) uptake of atmospheric CO_2_ might occur in green stems. Living tissues in bark (e.g. phloem parenchymatic and companion cells) and wood (e.g. ray parenchyma) as well as the stem cambium produce CO_2_ via respiration that might partially escape to the atmosphere, be partially re-fixed, but also dissolve in the xylem sap and thus contribute to xylem CO_2_. Depending on the ratio between the CO_2_ transport rate from the roots, stem efflux, stem assimilation and contribution of stem respiration to xylem CO_2_ transport, the xylem CO_2_ concentration might increase or decrease along the plant axis, but there is no information on acropetal xylem CO_2_ gradients available in the literature. The phloem might also transport CO_2_ but similarly no published data are available. In the leaves the efflux of leaf-respired CO_2_ plus the xylem-derived CO_2_ contribute to the total efflux. Stutz *et al.* (2017) showed that the efflux of xylem CO_2_ can approach 50% of the leaf respiration flux, but due to the difficulties of obtaining realistic twig-tip xylem concentrations and our lack of comprehensive data on night-time transpiration, generalizations might be difficult. Part of the xylem CO_2_ is fixed in the leaves in the night for anaplerotic reactions. Estimations of day-time respiration fluxes are notoriously difficult. During the day, leaf respiration rates might decrease due to substantial changes in leaf metabolism (Tcherkez *et al.*, 2009), but the contribution of xylem CO_2_ efflux might increase as transpiration rates are higher. If considerable amounts of xylem CO_2_ are fixed via photosynthesis, leaf-level photosynthetic measurements with classical gas-exchange measurement devices might underestimate leaf photosynthetic capacities. Note that the size of arrows does not indicate any quantitative differences in fluxes.

**Figure:**
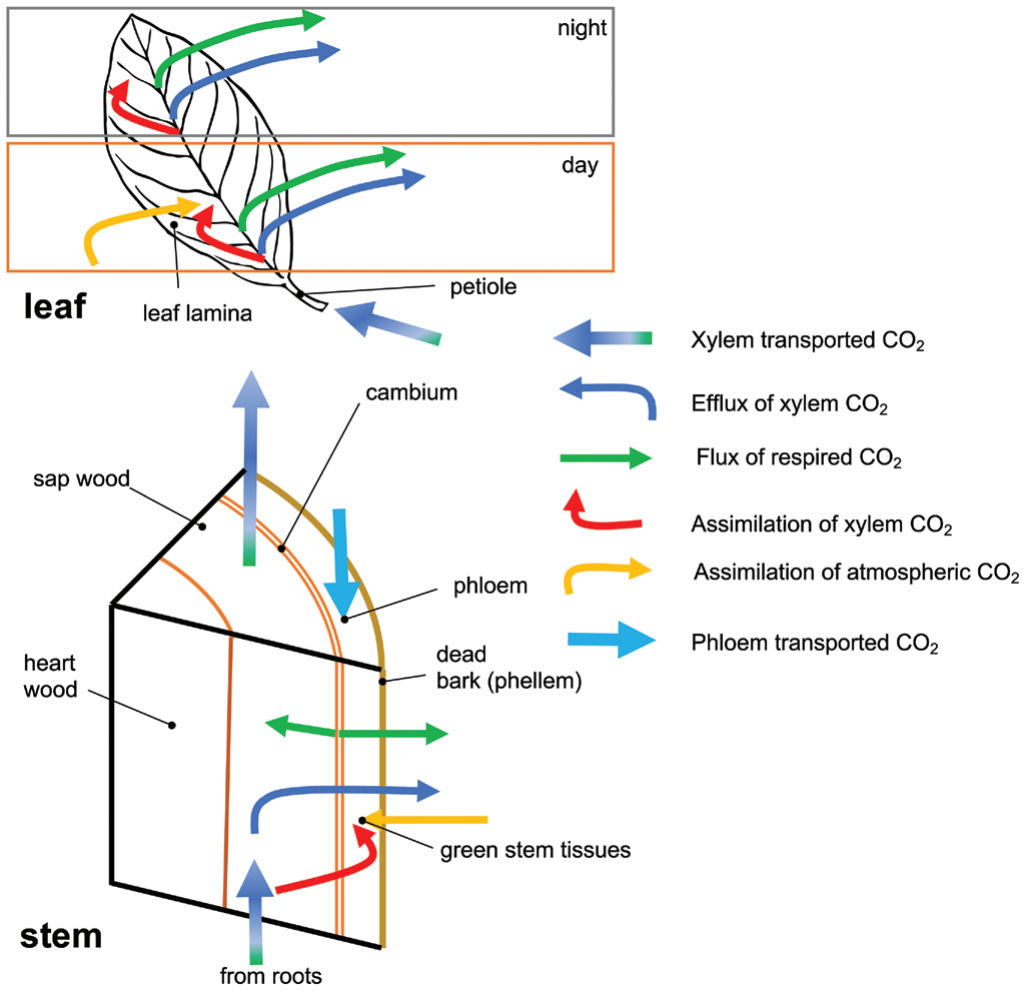

## Relationships between respiration and environmental drivers

Release of CO_2_ in plant tissues away from the original source of respiration does not necessarily affect the measurements of net ecosystem exchange or its separation into photosynthetic and respiratory fluxes. However, it might compromise a proper parameterization of respiratory CO_2_ fluxes from plant and ecosystem compartments which is needed to obtain a mechanism-based projection of ecosystem CO_2_ fluxes and thus the C balance of ecosystems under changing environmental conditions. For example, it is assumed that the temperature dependency of root and leaf respiration can differ (Atkin *et al.*, 2005). If, however, root-respired CO_2_ mixes with leaf-respired CO_2_ to form a combined efflux, which is affected by temperature *and* transpiration rate, such temperature dependencies cannot easily be separated. Moreover, estimates of assimilate distribution within the plant, and above- vs belowground C partitioning, will be wrong if we assume that up to almost 50% of root-respired CO_2_ is transferred via the xylem stream aboveground (Bloemen *et al.*, 2013), leading to a strong underestimation of the belowground C demand.

Stutz *et al.* (2017) conducted their measurements in the dark and concluded that mainly due to the low rates of night-time transpiration the efflux from xylem CO_2_ would on average amount to only about 1.5% of leaf respiration. There are, however, different uncertainties concerning this estimate. On the one hand, Resco de Dios *et al.* (2015) reported that nocturnal transpiration might be generally underestimated as it can contribute to >20% of day-time transpiration and thus the 1.5% might be a rather conservative estimate. On the other hand, there is no reliable information on the CO_2_ concentration in the xylem of twig tips or on concentration gradients from the main stem or branches, where a number of measurements exist (see Table 1 in Teskey *et al.*, 2008), to the twigs and petioles. If the assumption of Bloemen *et al.* (2013) that most of the xylem CO_2_ reaches the atmosphere via trunks and stems is correct, the CO_2_ concentrations reaching the leaves might be lower than the ones taken into account by Stutz *et al.* (2017), and thus they might overestimate the effective night-time flux. During the day, transpiration will be higher, but xylem CO_2_ concentrations vary inversely with xylem flow rates (Teskey *et al.*, 2008) and at least part of the CO_2_ approaching the leaf will be assimilated. Thus, we are far from being able to quantify the contribution of CO_2_ produced in roots and stems but released by the leaves. More research is needed to quantify xylem CO_2_ concentrations along the plant axis and changing with time, as are direct measurements of the efflux of xylem-borne CO_2_ via the leaf over diel timecourses.

## Leaf respiration in the light – old issues, new problems

During the light period, not only anaplerotic CO_2_ fixation, but also photosynthetic assimilation of xylem CO_2_, is likely to occur. Moreover, leaf respiration in the light is notoriously difficult to study. Only recently, Farquhar and Busch (2017) postulated that the Kok effect and the Laisk approach, which are usually applied, are error-prone and not straightforwardly applicable to estimate day respiration. Even though from a biochemical point of view it is well-known that the TCA cycle is not closed down in the light, and changes in the commitment of major biochemical pathways in the light and during light-to-dark transitions point to a reduction of CO_2_ release from glycolysis and the TCA cycle (Tcherkez *et al.*, 2009; Werner *et al.*, 2011), Farquhar and Busch (2017) suggest that it is currently best to assume that dark respiration in the light equals the respiration rate in the dark at the same temperature. If we now take into account that various amounts of xylem CO_2_ – depending on the variation of transpiration rate and of CO_2_ production, release and re-fixation in heterotrophic tissues – contribute to day-time CO_2_ efflux from the leaf, the situation gets even more complicated. Not only the complex and still poorly understood changes in leaf metabolism in the light, but also xylem-mediated teleconnections, will affect the measured CO_2_ fluxes. Moreover, net CO_2_ exchange measurement at the leaf level – an indispensable tool for plant physiology and ecology – will be incorrect if considerable amounts of the xylem CO_2_ are fixed, leading to underestimations of the real CO_2_-fixing capacity of a leaf.

## New challenges for understanding respiration – spatial and temporal influences on respiration

There is growing evidence that the CO_2_ efflux from aboveground tissues is affected by CO_2_ production from distant tissues connected via the xylem, and thus does not solely represent the local metabolism but rather the integrated plant activity weighted by the transpiration rate and the – still not sufficiently understood – assimilation in, and CO_2_ efflux from, different plant organs. And as if this situation were not complex enough in spatial terms, recent research indicates that temporal interconnections also affect leaf and canopy respiratory CO_2_ fluxes. Gessler *et al.* (2017) showed that in addition to the current environmental drivers affecting respiration, the antecedent conditions as mediated by the circadian clock were involved. This circadian control is assumed to act as an adaptive memory to adjust plant metabolism based on environmental conditions from previous days and thus adds a temporal component to respiration and its control. All these recent findings indicate that at least parts of our understanding of plant respiration have been too simplistic, but at least we do now have the tools in hand to fully account for spatial and temporal controls.
